# Football and Ice Hockey Fans' Experience of a 12-Week Training and Weight-Loss Pilot Intervention (ViSiT) in Sweden—A Focus Group Study

**DOI:** 10.3389/fspor.2021.616427

**Published:** 2021-08-18

**Authors:** Janna Skagerström, Magdalena Hjertstedt, Petra Dannapfel, Ulrika Müssener, Matti Leijon

**Affiliations:** ^1^Unit for Research and Development, Department of Health, Medicine and Caring Sciences, Linköping University, Linköping, Sweden; ^2^Unit for Human Resources, Department of Medical and Health Sciences, Linköping University, Linköping, Sweden; ^3^Department of Health, Medicine and Caring Sciences, Linköping University, Linköping, Sweden; ^4^Department of Medical and Health Sciences, Centre for Organizational Support and Development, Linköping University, Linköping, Sweden

**Keywords:** training intervention, weight-loss intervention, weight management, gender, masculinity, sports club, focus groups

## Abstract

**Background:** Excess weight is associated with an increased risk of poor health and premature mortality. This is more problematic for men than for women because men have a lower life expectancy and a higher prevalence of several lifestyle-related diseases. A concept whereby overweight male supporters of professional football clubs are recruited and offered a weight-loss intervention has been developed in Scotland. In the present study, we explore participants' experiences of a similar pilot intervention, called ViSiT, conducted with supporters in one ice hockey club and one football club in Sweden to assess the feasibility of using the intervention in a Swedish context.

**Methods:** In this user centered evaluation, focus groups were conducted with 12 men who had completed the 12-week ViSiT intervention. Participants discussed reasons for participating in and completing the intervention, effects of the intervention, advantages, and areas of improvement of the intervention, and thoughts on the club's involvement. The material was analyzed using thematic analysis according to Braun and Clarke.

**Results:** The analyses revealed four themes: reasons to participate, motivation and reinforcement, change of habit, and areas for improvement. The intervention was seen as an opportunity to change daily lifestyle behaviors. The group format, as well as the involvement of a prestigious sports club, was important for signing up to the intervention and for motivating continued involvement. The intervention had also resulted in increased knowledge on health and changed mindsets about being more attentive to regulating day-to-day behavior. Although the overall feedback on the intervention was positive, the participants suggested that possibilities to have more individual coaching should be added.

**Conclusions:** The ViSiT weight loss and lifestyle intervention may be feasible in a Swedish context to reach overweight men at risk of poor health. The ice hockey and football club supporters expressed similar experiences from participating in the intervention. ViSiT seem to have a potential to be adopted by many sports clubs for a widespread reach to a group normally considered reluctant to participate in lifestyle change programs.

## Introduction

Excess weight is associated with an increased risk of poor health and premature mortality. Obesity contributes to increased risk of all-cause mortality worldwide, with higher risk in men compared to women (The Global BMI and Mortality Collaboration, 2016). In Sweden, 57% of men were overweight or obese [body mass index (BMI) >25 kg/m^2^] in 2016, and the prevalence is increasing in all age groups except those aged 65–85 years (Folkhälsomyndigheten, 2017).

Traditionally, men are underrepresented in community-based weight-loss intervention studies (Pagoto et al., 2012) and in workplace health promotion interventions (Robroek et al., 2009). Also, men appear to be less knowledgeable about risk factors for health and more reluctant than women to engage in health-promoting programmes (Pagoto et al., 2012; Gray et al., 2013; Carroll and Lambe, 2014; Ek, 2015). On this point, there have been several recent initiatives assessing gender-sensitized healthy lifestyle interventions for men performed in contexts such as their work place, with their children or technology-based (Caperchione et al., 2016; Seaton et al., 2017; Williams et al., 2018; Young and Morgan, 2018). Sport venues have been shown to be good locations for gathering people with low health literacy that are not easy to reach with health interventions and health education (Drygas et al., 2013). A new method of attracting men who need to lose weight, a concept called football fans in training (FFIT), has been developed and studied in Scotland. FFIT aims to recruit overweight supporters of professional football clubs and offer them a weight-loss intervention combining evidence-based behavioral change techniques to improve physical activity, sedentary time and diet.

The FFIT intervention uses the traditionally male environment of football clubs, and the powerful social and psychological connections to the team (e.g., belonging, identity, loyalty, validation) that being a supporter creates (Hirt and Clarksson, 2010) along with the opportunity to participate in a men-only group, to maximize men's engagement with an evidence-based, gender-sensitized weight management programme (Wyke, 2012). The FFIT has been designated best practice to affect the lifestyle of the target population by the Canadian Public Health Agency. FFIT has been shown to attract men who were at very high risk of ill health (Hunt et al., 2014a) and shows good results on weight loss (adjusted mean difference between intervention and control 4.9 kg) (Hunt et al., 2014b).

FFIT has been conducted and evaluated in Great Brittan where the football culture is strong, and the football club reaches a large number of people (Ireland and Watkins, 2010; Drygas et al., 2013). In Sweden the highest league in football (Allsvenskan) is followed by the highest league in ice hockey (SHL) when it comes to attendance of spectators. For Allsvenskan the average audience attendance is just over 9,000 spectators which is Europe's 17th most popular league. For SHL the average audience attendance is about 6,000 spectators. To our knowledge there is no research available on the feasibility of interventions similar to FFIT in countries with a smaller supporter population. Therefore, we have developed an intervention inspired by FFIT and pilot tested it in a Swedish setting (Leijon et al., 2019). The intervention is called ViSiT (Swedish acronym: VIktiga Supportrar I Träning) and consists of a 12-week group-based lifestyle programme with weekly group sessions, followed by a 9-month maintenance phase. The aim of this study was to explore the participants' experiences of the intervention and thereby assess the feasibility of using the intervention in a Swedish context.

## Materials and Methods

### Design

In this user-centred evaluation of the ViSIT-intervention, data were collected through focus groups with participants in the ViSiT project. Focus groups allow interaction between the participants and offer the potential to gather information on diversity or consensus within the group (Krueger and Casey, 2009). The study was performed according to the World Medical Association Declaration of Helsinki ethical principles for medical research involving human subjects. The study was approved by the Regional Ethical Review Board in Linköping (Dnr 2016/50-31).

### Study Setting

The intervention study was set in south east Sweden. Supporters for the intervention were recruited from two sports clubs, one football club and one ice hockey club, both in the highest national league. Both clubs have well-attended matches and active supporter groups. Information on the intervention was posted on the clubs' websites, Facebook sites and was also part of an e-mail sent from the club to members. To increase motivation to participate the intervention was described as a chance to learn about physical activity, healthy eating and motivation from professionals in the non-profit sport organization (Östergötlands Idrottsförbund) and from the sport club. The invitation was signed by the club director and representatives from the non-profit sport organization as well as the PI researcher (ML). Inclusion criteria for taking part in the intervention were men aged 35–65 years, BMI >28 kg/m^2^ [the same criteria as used in FFIT and for an evaluation of Hockey Fans in Training in Canada (Gill et al., 2016)].

### ViSiT Outline and Measurements

The ViSiT intervention program was designed and conducted to engage overweight and obese male supporters to lose weight through improved physical activity and lifestyle changes. The intervention consisted of 12 group sessions consisting of a theoretical lecture (see Table 1) (~60 min) and 30 min of physical activity; the duration and intensity of the physical activity increased over time. Activities included both cardio and resistance training such as brisk walks, stairclimbing in the arena, circuits with body weight exercises and training-equipment as well as traditional workout-sessions in the stadia fitness center/gym. The group sessions were held in facilities connected to the club, either the club stadium (for the football club) or a gym owned and used by the club (for the ice hockey club). A project manager from the Östergötland Sports Federation along with a public health practitioner planned and performed the lessons. Employees from the clubs such as the fitness coach, mental trainer and nutritionist were enrolled in some elements to further strengthen the link to the club and increase the participants' motivation to attend each session in order to learn from several different professionals. The concept was based on the concept reported in the FFIT studies and especially Gray et al. (2013) concerning the development and optimization of the FFIT intervention and included components related to, for example, healthy eating, physical activity, alcohol, goal setting, motivation, and relapse prevention. The participants were also offered a pedometer to use during the intervention. All participants were encouraged to share pedometer data with the others via a mobile phone application for social support as well as for motivation. Following on from the 12-week intervention, a 9-month maintenance phase consisted of six post-program e-mail prompts and a group reunion at the club 6 months after the last of the 12 sessions. One intervention group per club was held in spring 2016. The groups consisted of 8 and 14 members, respectively, with attendance rates of 88 and 86%, respectively, for the group sessions.

**Table 1:** The 12-week intervention program.

**Week**	**Activity**	**Content**
1	Introduction and anthropometry	Presentation of participants and program coordinators, goal-setting, rules for the group work, introduction to pedometers, short walk.
2	Food and drinks	Diet and smart goals, introduction of 3-days food diary, 15 min walk.
3	Foods and health labeling	Feedback on food diary and healthy dietary changes, understanding food packaging labels, 30 min physical exercise.
4	Physical activity and implementation	Importance of physical activity to health and well-being, sedentary time, action planning for physical activity, 30 min physical exercise.
5	Energy in drinks	Alcohol, other drinks and weight gain, 30 min physical exercise.
6	Half way anthropometry	Performance feedback, body weight and compositions measurement, meeting club player and trainers, 30 min physical exercise.
7	Sports psychology	Feedback on groups achieved weight-loss, sports psychology (motivation, confidence, mindset, and behavior), 30 min physical exercise.
8	Health benefits and implementation	Health benefits associated with weight loss, role of social support, 30 min physical exercise.
9	Healthy choices	Make favorite food healthier, make eating out meals healthier, 30 min physical exercise.
10	Sports psychology	Sports psychology (emotions, behavior, relapse, triggers, locus of control, identity) homework 3-days food diary, 30 min physical exercise.
11	Recapitulation and future perspectives	Feedback on food diary, future strategies and goal-setting, 30 min physical exercise.
12	Closure and anthropometry	Performance feedback, questionnaires, body weight, and composition measurement, relapse prevention, ongoing social support, information and planning for continuation (e-mail-prompts every 6 week, reunion after 3 months), 30 min physical exercise.

### Procedure

At the first group session, the participants were informed that focus groups would be taking place 1 week after the 12-week intervention ended. All participants who completed the intervention program were asked to take part in the focus groups Participants who dropped out of the intervention at an early stage were not invited. Twelve participants from the two intervention groups (in total 22 participants) were eligible for the focus groups. Three focus groups were held, two with football supporters and one with ice hockey supporters. The football supporters were randomly divided into two groups with four participants in each. The focus groups were conducted in June 2016 and lasted for 30–59 min (average, 49 min). Before the focus groups started, the participants were asked to once again read a letter containing information on the study, stating that participation was voluntary, that the results would be reported so that the participants cannot be identified and that the focus groups would be recorded. The participants had left their consent to participate in the group interviews at the same time as they registered to participate in the intervention. At that time the consent was left by clicking a link at the internet. At the time of the interview the participants also gave their verbal consent.

A semi-structured interview guide was created for use in the study. The interview guide contained questions on reasons for participation in the intervention, important effects of the intervention, what made the participants complete the intervention, the participants' perceptions about the success of the intervention, areas for improvement of the intervention and thoughts on the club's involvement in the intervention. Probing questions were used to direct the conversations and get the participants to give detailed answers. Before starting the focus groups the moderator and observer introduced themselves, their role in the research project and interests in the area. In order to make the participants feel relaxed, and to make sure that everyone said something, the first introductory question concerned the best game so far this season for the team they supported. The focus groups were held at the same location as the intervention.

In order to test the interview guide and acquaint the moderators with their role, three pilot focus groups were conducted with men who had gone through the ViSiT intervention in a previous round. Minor revisions were made to the interview guide. The pilot focus groups were not analyzed for this study. All focus groups were moderated by a researcher (Ph.D.) with insight on the project and experience of conducting focus groups; another researcher (Ph.D.) acted as observer. One of the researches was male and one was female and none of them had any relationship to the participants in the focus groups. The role of the moderator was to ask questions and lead the discussion forward. The observer took care of practical issues, took notes and followed up on specific areas at the end of the focus groups. No one but the researchers and participants were present at the focus groups.

### Data Analysis

Interviews were recorded digitally and later transcribed verbatim. The data were analyzed using thematic analysis according to Braun and Clarke (2006). The analyses were based solely on the transcripts; body language and interaction between the participants were not taken into consideration when conducting the analyses. Thematic analysis is a technique for analyzing texts based on empirical data to find repeated patterns of meaning. It can be used to report experiences of participants (Patton, 2002). The aim of this study was not to unravel a deeper meaning on the phenomenon investigated; rather, the focus was to reflect the participants' experiences (Patton, 2002; Braun and Clarke, 2006) and to understand the participants' reasons for participating and following through with the ViSiT intervention. Thematic analysis entails a structured process to code and categorize the data. Themes and patterns are identified with an inductive, data-driven approach. The analysis was performed in steps, as suggested by Braun and Clarke (2006):

First, each author read all the transcripts separately to obtain an understanding of the whole material. After reading the whole material, JS and PD went through the transcripts several times and started to analyse and search for meanings and patterns that described the essence of the respondents' statements. After initial familiarization of the whole dataset, data were coded systematically. Data that reflected various key statements and thoughts in relation to the study's aim were sorted into initial codes and labeled based on the core content. After initial coding of the dataset, the codes were re-analyzed, focusing on how the codes could be interrelated and if they described the same phenomenon; from this analysis overarching themes were formed. During the process, JS and PD independently analyzed and coded the same data and then compared findings and discussed similarities and differences in interpretation of the data. The initial themes were refined by rereading the data to confirm that the codes collected to construct a theme had a coherent pattern and/or enough data to be considered a theme. The proposed themes were then reviewed in relation to the entire dataset. All authors discussed the content of the themes using triangulating analysis. This discussion continued until no inconsistencies existed and a shared understanding was reached (Braun and Clarke, 2006).

The themes were reviewed with regard to what they added to the reporting of the data. Subthemes were identified and themes were defined and named.

Quotations illustrating the essence of the themes were chosen and translated from Swedish to English; all authors collaborated in the translation. The quotations were then embedded in an analytic narrative that included the overall scope of the theme.

## Results

In total 12 men who had participated in the ViSiT intervention were in the focus groups (3–5 participants per group). Eight were married or cohabiting and four were single. Among the men who participated in the focus groups, three had children living at home; 10 out of 21 of the participants in the intervention had children living at home. Nine of the men were working. For further information on the participants in the focus groups and the men in the ViSiT intervention is given in Table 2.

**Table 2:** Baseline characteristics of all participants from the second term and those who took part in the focus group.

**Variables**	**Total (*n* = 21), *n* (%)**	**Among those who took part in the focus group (*n* = 12), *n* (%)**
**Marital status**
Married/living together	17 (81.0)	8 (66.7)
Single	4 (19.0)	4 (33.3)
Children living at home	10 (47.6)	3 (25.0)
Born in Sweden	20 (95.2)	11 (91.7)
**Education**
Primary	3 (14.3)	2 (16.7)
Secondary	12 (57.1)	7 (58.3)
University	5 (23.8)	3 (25.0)
Other	1 (4.8)	0 (0.0)
**Occupation**
Working	18 (85.7)	9 (75.0)
Disability pension	2 (9.5)	2 (16.7)
Other	1 (4.8)	1 (8.3)
Monthly income (in SEK)	(*n* = 20)	(*n* = 11)
<25,000	4 (20.0)	2 (18.2)
25,000–30,000	7 (35.0)	3 (27.3)
30,000–35,000	4 (20.0)	4 (36.4)
>35,000	5 (25.0)	2 (18.2)

Based on the conversations between the moderator and observer after the focus groups, a general observation was that the atmosphere in the groups was relaxed and open. It was clear to the researchers that the participants had come to know each other well-enough to share their thoughts in the focus group situation. Analysis of the data yielded four themes that the participants discussed in relation to the ViSiT intervention: reasons for participating, motivation and reinforcement, change of habits, and areas for improvement.

### Reasons for Participating

Before starting the intervention, the respondents saw several potential advantages that made them interested in participating. Further, they knew that they needed to do something about their unhealthy lifestyle as they were aware that their overweight and inactivity was associated with risk for disease.

#### Opportunity to Change

Before the study the respondents were inactive and overweight and they stated that they had little knowledge about physical activity and nutrition. Participation in ViSiT was perceived as an opportunity to get good advice regarding exercise and eating habits. To participate in ViSiT was an active choice by the respondents and functioned as a “new start” for the participants.

“*To get some more professional help. To really learn what to eat and how much, that has been very hard before, to know how much to eat.” (Group 2 participant 2)*

“*I called her and she told me that I was selected to participate. I felt this is the last chance and I have to take it so I had high expectations of the program; that's why I was really serious when we started.” (Group 1 participant 2)*

#### Being Part of a Homogeneous Group

Another important motivational factor was to be able to do this in a group with men similar to themselves. The knowledge that all participants were overweight and in need of the intervention made the respondents feel more comfortable with the intervention, in particular the physical activity. Some respondents mentioned that earlier attempts to start exercising at a gym or at classes open to anyone had made them feel like outsiders because they felt unfit and in bad shape compared with others. On the other hand, a few participants stated that they did not think that it would have a negative impact if younger men as well as women were also included, as long as they were supporters and overweight.

“*I could do it over again with you or with some likeminded people in a group like that, but not a regular gym. I tried that once, never again.” (Group 1 participant 2)*

“*I wouldn't mind some women joining. I was more surprised that there were no women here when I got here. Because it (the recruitment information) didn't say only men could apply*.

*Yes it did, it said men between 35 and 35 and BMI over 30.” (Group 3 participant 1 and 3)*

#### Club Involvement

Being invited by the club that many of the supporters had followed for several years brought an extra dimension to the intervention. Some informants would not have participated if it were not for the involvement from the club. To use club facilities and especially to meet representatives from the club provided that extra inspiration to participate. By participating in ViSiT the men were seen by the club and that their relationship with the club became more personal.

“*I believe that the program is cool, you do have feelings for the club. Just to be in the facilities and exercise and maybe get to talk to some of the players is really motivational.” (Group 3 participant 5)*

“*… that it was the club, that is in all our hearts, that is behind this is an extra drive and I think that that contributes a lot to all of us being here.” (Group 3 participant 1)*

### Motivation and Reinforcement

Dropping out was not something the participants thought of once the intervention had started. Rather, there were several factors that sustained their motivation during the 12 weeks. Several participants mentioned that they could have kept going in the same manner for a longer period of time.

#### Visible Results

To see results of the efforts made during the intervention was encouraging for the participants. They mentioned seeing results both in relation to weight loss and to improved fitness level and mental health. Feeling that their clothes were less tight, having to change to a smaller size or getting compliments about losing weight were examples of reinforcing events related to losing weight. Not getting breathless as much as before and feeling more alert and positive were other results that the participants experienced during the intervention. It was expressed that noticing these effects, mostly in one self but also among others in the group, kept the motivation and inspiration going throughout the intervention.

“*My kids don't live at home anymore but when they saw me after a couple of weeks it was like, “wow! What have you done? That's so good!” Just positive comments, it was almost the best part.” (Group 1 participant 1)*

“*Before when I tied my laces, I had to stand up and breathe between each shoe, now I can tie them both without breathing.” (Group 3 participant 2)*

#### Fellowship and Solidarity

Being in a group made exercise easier as all participants had the same goal and could support each other. It was found to be more fun and inspiring to do this together with other likeminded people with similar prerequisites.

“*We were about as heavy, we like football, we shared the same interest and have something in common, you can talk about football.” (Group 2 participant 3)*

The activities and progress other members worked as a motivator to continue. Being part of a group gave that extra kick and push. Further, it was discussed in the groups that fellowship motivates and makes it fun to attend the meetings.

“*It's that feeling of fellowship and pep and also that boost to help you get going.” (Group 2 participant 1)*

“*It's fun to hear what the others in the group are struggling with and how they handle it and what they did to keep up their motivation. You get more energy from it, from talking to the others in the group because you are going through the same things.” (Group 3 participant 3)*

Further, knowing that the club had invested in them created a willingness to fight hard and succeed with weight loss. Being successful with weight loss was suggested to be a way to give something back to the club. Because the club had offered the opportunity to participate in ViSiT free of charge, the respondents could not let the club down.

#### Comparison

Not only did the results of others serve as a source of motivation, it was also said to trigger competition. The feeling of competition was intrinsic and described as not wanting to be below average when it came to the physical activity level or amount of weight loss.

Being controlled or informally checked by the group and the leaders was believed to strengthen the motivation to keep up the work and efforts week by week. The results were related to the formal parts of the concept, such as the half-time weigh-in, the weekly report from the pedometer, and to the more informal check by the peers in the group. To be able to follow your own progress and compare it with the other group members kept up the motivation, and for some respondents also helped them to increase their efforts.

“*The weigh-in at half-time was good; that gave me a great boost. You had lost weight and got even more triggered.” (Group 1 participant 3)*

“*I need to be hounded, meet someone I can talk to; to run alone is so boring.” (Group 2 participant 2)*

As well as the internal competition within the groups, the respondents revealed an encouraging rivalry among the supporters with the other weight-loss groups. The football supporters wanted to lose more weight at group level than the ice hockey supporters and vice versa. The participants in the program conducted in spring 2016 also compared their group results with pilot-project groups from 2015 (not part of the present study).

“*For me, the little competitive feeling toward the other group in Linköping (the other city) and earlier groups that you want to beat and your own goal trigger me.” (Group 3 participant 1)*

### Change of Habits

#### Knowledge to Make Healthier Decisions

With increased awareness and motivation regarding nutrition, the respondents started to change their food choices. They expressed that the information given in the intervention had been available to them before starting the intervention, but they had not absorbed such information from newspapers, TV shows, etc. When the information was addressed directly to them, it was easier to transform the information into practical knowledge when making food decisions.

“*Before I thought that I should not eat anything but then that's starvation and really boring. But one can indulge in reasonable amounts and also think a little more critically about what you buy, turning over the packaging and thinking that there might be a bit too much carbohydrate in this and take another product instead. In the past, things were just grabbed off the shelf out of habit.” (Group 1 participant 4)*

The respondents also had an impact on people in their surroundings as they spread their knowledge and motivation to change habits. Family and colleagues were impacted by their dietary changes in direct or indirect ways. For example, family members often ate the same as the respondents, having a direct impact on them. With colleagues, the impact was more indirect as they became inspired by the healthy lifestyle, or even by the visible weight loss, of the respondents.

“*My wife eats the same as I do. I think she has tried to encourage me in the background before, but since I became redeemed, it is much easier, and we have both changed our eating habits. I eat salad five times a week, my sons never would have believed that. I have also inspired some people at work.” (Group 2, participant 3)*

#### Different Mindset

The intervention gave the respondents a new way to think and make decisions. Breaking behavioral patterns and implementing a new lifestyle that would remain after the intervention was a recurring topic of discussion. The respondents voiced that they had started thinking differently and prioritizing about being healthy; they were no longer looking for a quick fix to lose weight. They made jokes about how unaware they had been about lifestyle issues before starting in the ViSiT group, implying they would not make those choices again. They gave several examples of strategies they had created to avoid drawbacks when being in situations where they used to consume a lot of alcohol or indulge in snacking or eating unhealthily.

“*How you attack things and how to act in situations, like don't go shopping when hungry.” (Group 1 participant 2)*

“*Like if you drive on the motorway and stop at a petrol station, now I buy something healthier. Before I bought candy and put the bag in the seat beside me and ate.” (Group 2 participant 3)*

### Areas for Improvement

In general, the respondents were happy with the intervention and thought they were lucky to have been included. However, they had some suggestions on improvements for future groups.

#### More Time for Physical Activity

One recurring suggestion was to increase the time spent on physical activity. As the target group was not very used to working out it took a long time for them to learn different exercises why more time could be needed. One solution to this problem that was raised was to provide instructional films that the participants could watch at home as a complement to the instructions given at the group sessions. Some parts of the physical activity lessons were designed to be instructive and inspiring rather than an intense workout, which was disappointing to some participants.

“*I feel that some training sessions were too short. At least that is how I felt. Short but intensive. Yes, but not intense enough. I feel that some workouts could've been a bit more.” (Group 1 participant 1)*

#### Individual Feedback

Another request for future interventions was more personalized feedback. Several respondents would have liked to talk to someone about their own eating habits, activity level or struggles rather than just having general information. The individual feedback that was provided in connection with the three weigh-ins was appreciated and there was a wish for more individual feedback. Further, several participants struggled with injuries (both traumatic and overuse) and would have appreciated individual advice on how to proceed or how to avoid getting hurt when exercising.

“*It would have been good to have access to a physiotherapists or someone with knowledge in rehabilitation /…/ since I don't think that any of us have been free from injuries.” Group 3 participant 2)*

## Discussion

The aim of this study was to explore sports club supporters' experiences of the ViSiT intervention. Our results indicate that this type of intervention is feasible in a Swedish context, in both football and ice hockey clubs, with a general positive attitude among the participants.

The original FFIT study was carried out in Scotland where football plays an important role in the male culture, and the sport and club context provides an opportunity to reach a large number of men with less favorable health behaviors (Drygas et al., 2013; Hunt et al., 2014a). A similar intervention, Premier League Health, has been carried out in England where the football culture is also strong (Pringle et al., 2013). An adapted version directed to ice hockey fans is carried out in Canada where ice hockey is an important part of being Canadian (Gill et al., 2016). In Sweden, football and hockey are the predominant sports in terms of the number of players and supporters. However, the culture is not as pronounced as in Scotland and Canada. Still, the national top leagues within football and ice hockey are large enough to have an intense supporter culture with loyal members. The results from this study show that the concept of lifestyle interventions promoted by sports clubs seems to attract the target population in Sweden. The participants agreed that both the club involvement and the fact that the intervention was directed to a homogeneous group made them sign up and helped maintain their motivation. From the focus groups, we could not see any differences between the supporters from the football club and ice hockey club. These results are similar to results from FFIT showing that the setting and social situation with likeminded men encouraged the participants and contributed to the desirability to participate in the intervention (Hunt et al., 2014a; Bunn et al., 2016). It has been suggested that associating a health behavior intervention with the commitment and pleasure of being a fan can turn motivation to support the team into motivation to lose weight together with fellow fans (Duncan et al., 2012).

The participants in this study mentioned the importance of the program being arranged by the clubs. The fact that the invitation was sent by the club seemed to make the men consider participation and made them feel addressed and selected in a direct manner. Cialdini's (2007) work on influence and some of the six principles of persuasion could help explain the importance of the club engagement. For example the principle of *liking* states that if people like someone they are more prone to say yes, the principle of *authority* says people defer to authorities and experts, the principle of *commitment and consistency* states people will try to follow through if they make a public, voluntary commitment and the principle of *scarcity* explains that people value things more in the consider that they are scarce. In a review of 31 intervention programs to promote physical activity for adult men, including FFIT studies, it was concluded that all studies using professional clubs to recruit men resulted in increased physical activity (Bottorff et al., 2015). Further, the ViSiT program resulted in clinically relevant weight reduction for the participants (Leijon et al., 2019).

In several studies, such as POWER, ManUP, FFIT and POWERPLAY, a sports club or a male workplace setting has been suggested to facilitate men's engagement and to be suitable to combine with a gender-sensitive approach (Duncan et al., 2012; Morgan et al., 2012; Gray et al., 2013; Gill et al., 2016; Seaton et al., 2017). Lozano et al. (2016) suggest that overweight men's fear of being stigmatized might keep them from places such as public gyms, but can also simulate participation in weight management interventions (Lozano et al., 2016). Men in our focus groups mentioned being comfortable among equals, however, a few would not mind if the group included women and younger men. This could reflect that, for some participants, the connection to the club was more important than feelings of homogeneity in the group. Data included in a review study could not establish if male-only environments were preferable to men as in a few studies the male-only features were less important than other factors (Robertson et al., 2014).

The attendance rate in ViSiT was high for more information see (Leijon et al., 2019). and the participants mentioned that they did not want to miss any of the sessions. The familiar setting at the club as well as identification with the other participants was found to be important for FFIT as well as the Premier League Health (PLH) program (Robertson et al., 2013). Evaluations of both programs have highlighted the importance of providing a context that matches the identity and values of the supporters (Robertson et al., 2013; Hunt et al., 2014a). Bunn et al. (2016) highlighted that to develop acceptable weight management interventions, one needs to be aware of relevant social practices if behavior change is to be sustainable. Further, Lozano-Sufrategui et al. (2018) recommend that health promoting intervention prioritize the social aspects within the intervention for more sustainable health improvements. The same seems to be valid for ViSiT, because the men reiterated the importance of the club involvement. The possibility to meet the players and important persons in the club and seeing the club from the inside seemed to have played a large role in removing barriers associated with a weight-loss intervention. Sociality and interaction within these settings play an important role in facilitating a behavior (Bunn et al., 2016). Further, (Robertson et al., 2013) stress positive social interaction as a central mechanism for further engagement and as a facilitator for changing one's behavior.

The exercise sessions held with the group were all supervised and attended by the leaders. In the review by Bottorff et al., 2015, programs including supervised exercise sessions seemed to be more effective in increasing physical activity than programs relying on self-monitoring. In the results from ViSiT, the men mentioned that they, as a group, were not used to doing different physical exercises and that it took a long time to learn new exercises. This might be a reason why supervised exercise sessions are more effective. The men wished for more time to learn the exercises in the group and to have instructional videos to watch at home to make sure that they performed the exercises correctly. Earlier research has indicated that simplicity and clear rules regarding, for example, healthy eating might be a success in interventions targeting men (Collins et al., 2011; Morgan et al., 2012; Seaton et al., 2017). Bearing in mind that men are more reluctant than women to engage in health-promoting programs (Robroek et al., 2009; Pagoto et al., 2012; Gray et al., 2013; Ek, 2015), there is a possibility that perceived complexity in physical activity and/or advice regarding eating acts as an inhibitor to attend such programs.

The participants were monitored and individual results from weigh-ins were discussed openly among the group members. Some participants seemed to turn the open sharing of results into a comparison and reported they were encouraged by the competitive spirit. According to Social Cognitive Theory (SCT) (Bandura, 1986), individuals can benefit and learn from others' actions. Based on someone else's outcome from performing a behavior, the individual might decide to perform or not perform the specific behavior. Further, in SCT, expectations regarding social reactions to a behavior are part of the decision to change a behavior. Applying SCT on ViSiT, it can be assumed that some men were inspired and motivated by seeing the weight loss of other men. Seeing the efforts made by others and the resulting weight loss could be a trigger for some men to enhance their own efforts. This kind of extrinsic motivation (behaviors are performed to obtain a certain outcome) has been found to be less associated with long-term behavior change than intrinsic motivation (i.e., when the behavior is performed for the inherent pleasure in performing it) (Ryan et al., 1995; Conner and Norman, 2005). Gaining competence in activities related to a behavior facilitates this transition, and getting positive feedback on a behavior can enhance the intrinsic motivation (Deci, 1971; Vallerand, 1997). In ViSiT, the leaders worked on supporting, giving positive feedback and most importantly providing skills and competencies about eating habits and physical activity. Hopefully, this led to an increased level of intrinsic motivation among the participants, which may keep them on the same track after the end of the intervention.

Although the participants mentioned positive aspects of openly sharing results and being in a competitive environment triggered them to perform at a higher level, there was a wish for more individual feedback, individual advice and in-depth conversations with the leaders. Here, the competitive and light-hearted atmosphere of the intervention might be a hindrance whereby it is hard to talk about more sensitive or personal issues. Further, the open sharing of results could potentially be disapproved of by men who are less competitive by nature. Sports settings are intimately tied to constructions of masculinity in many cultures (Connell and Messerschmidt, 2005) and, according to de Visser and McDonnell (2013), weight-loss concepts created for men in a manly environment may appeal to men's feelings of masculinity. Men are more likely to engage in health risk behaviors, potentially explained by gender role socialization which encourages men to take more risks when it comes to health (Courtenay, 2000). Further, construction of masculinity as being self-reliant rather than help seeking is another possible explanation (Mahalik et al., 2007). Men's health behaviors are embedded in a social context, including normative group behaviors guiding what behaviors are deviant or off limit. Groups that are regarded as being similar to oneself are most influential. Perceptions of normative health behaviors in other men may have a powerful influence on an individual's adoption of health behaviors (Mahalik et al., 2007). Several men in this study stated that the intervention gave them a new way of thinking and that they thereby broke behavioral patterns. Further, they mentioned that their new way of life had an impact on people in their surroundings, i.e., their colleagues. The respondents in ViSiT could act as ambassadors for new health norms.

The FFIT program is now in use in all 13 Scottish Premier League clubs, reaching hundreds of men annually since 2010 (Bunn et al., 2016). The potential of reaching a large number of men using interventions like FFIT is encouraging because this group has traditionally been hard to reach and there is a call for more interventions directed toward men (Hunt et al., 2014a). Although the supporter population in Sweden is small compared with other countries, the initiation of ViSiT shows promising results. So far, four groups (the two included in this study and two pre-study groups not included here) have been held within the ViSiT concept. The two clubs involved have shown interest in proceeding with more groups. In Sweden, 57% of men and 44% of women were overweight or obese (BMI >25 kg/m^2^) in 2016 (Folkhälsomyndigheten, 2017). If the supporter culture is shared in all clubs and if the concept was implemented in all football and ice hockey clubs in the highest division (16 football teams and 14 ice hockey teams), hundreds of men in the current target population could be reached by the intervention annually. Further, there is a possibility to broaden the target population to include younger men, men with lower BMI or to try female groups to reach even more supporters. Non-profit sport associations as well as sports clubs play an important role in the promotion of physical activity and sports during the lifespan. The Swedish Sports Confederation has presented a new Sport for All strategy that includes lifelong involvement in sport and a clear vision about the importance of sport organizations as a part of society (The Swedish Sports Confederation, 2016).

Based on the results of this study, we recommend that future interventions add some more personal feedback to the participants who request that. This personal feedback might be a way to further increase motivation. Another recommendation is to keep physical activity exercises quite simple at the group sessions as there is limited time. More advanced exercises could be offered to those who request it, both at the group sessions and as home-based exercises to conduct between group sessions. Further, research on the effectiveness and cost-effectiveness of the intervention in a Swedish setting are needed before a large-scale intervention is planned.

The study has some limitations that should be considered when interpreting the results. As we recruited fans through the club website and e-mail lists, we cannot estimate the reach of the intervention. Further, the recruitment strategy could have led to inclusion of the most motivated men, which is why the results might be overly positive. Although the same recruitment strategy is planned in the future, the men who signed up for the first round might differ from those signing up in the future with regard to motivation, readiness for change, etc. In addition, as this concept was new in Sweden, it got a lot of attention from the mass media, which could have increased the motivation among the clubs and the participants, leading to extra efforts from all involved. Further, all qualitative research is limited regarding generalizability of the results to other settings and populations. All groups form their own climate and norms regarding behavior, trust and level of sharing, which may also affect the generalizability of results. In qualitative research, it might be more appropriate to discuss the concept of trustworthiness. We used triangulation to address the credibility of the study. Further, we believe that the results might be transferable to similar populations and applicable in contexts similar to ours. The analysis has been kept close to the respondents' narratives to avoid researcher bias. To strengthen the dependability, we have strived to be explicit regarding the content of the intervention and the procedure for this study.

The project manager and public health practitioner delivering the intervention (coaches) were also involved in the planning of the intervention, and therefore they had a deeper knowledge about the concept than could be expected from coaches delivering the intervention within one club. The coaches' dedication toward the intervention might have reduced the transferability of the results to other clubs.

The reporting of the study follow the Consolidated criteria for reporting qualitative research (COREQ) which is a 32-item checklist for interview and focus groups studies (Tong et al., 2007).

## Conclusions

In conclusion, the ViSiT weight loss and lifestyle intervention may be feasible in a Swedish context. The involvement of the sports club and the opportunity to be in a group with other men in a similar situation were reported to be important for joining the intervention and in promoting motivation during the intervention. Although the intervention was a group activity, the participants appreciated the individual feedback they got in relation to, e.g., weight-loss results. The ice hockey and football club supporters expressed similar experiences from participating in the intervention. ViSiT has the potential to be adopted by many sports clubs for a widespread reach to a group normally considered reluctant to participate in lifestyle change programs.

## Data Availability Statement

The raw data supporting the conclusions of this article will be made available by the authors, without undue reservation.

## Ethics Statement

The studies involving human participants were reviewed and approved by Regional Ethical Review Board in Linköping (Dnr 2016/50-31). Written informed consent for participation was not required for this study in accordance with the national legislation and the institutional requirements.

## Author Contributions

ML, JS, and MH designed the study. JS and ML conducted the interviews. PD and JS undertook the initial analysis and interpretation of the data, which was followed by discussions with ML, MH, and UM. All authors drafted the manuscript and were part of the revision process and agreed to the final version of the paper before submission.

## Conflict of Interest

The authors declare that the research was conducted in the absence of any commercial or financial relationships that could be construed as a potential conflict of interest.

## Publisher's Note

All claims expressed in this article are solely those of the authors and do not necessarily represent those of their affiliated organizations, or those of the publisher, the editors and the reviewers. Any product that may be evaluated in this article, or claim that may be made by its manufacturer, is not guaranteed or endorsed by the publisher.
